# Bio-inspired Construction of Advanced Fuel Cell Cathode with Pt Anchored in Ordered Hybrid Polymer Matrix

**DOI:** 10.1038/srep16100

**Published:** 2015-11-05

**Authors:** Zhangxun Xia, Suli Wang, Luhua Jiang, Hai Sun, Shuang Liu, Xudong Fu, Bingsen Zhang, Dang Sheng Su, Jianqiang Wang, Gongquan Sun

**Affiliations:** 1 Division of Fuel Cell & Battery, Dalian National Laboratory for Clean Energy, Dalian Institute of Chemical Physics, Chinese Academy of Sciences, Dalian 116023, China; 2 University of Chinese Academy of Sciences, Beijing 100039, China; 3 Shenyang National Laboratory for Materials Science, Institute of Metal Research, Chinese Academy of Sciences, Shenyang 110016, China; 4 Fritz Haber Institute of the Max Planck Society, Faradayweg 4–6, 14195 Berlin, Germany; 5 Shanghai Synchrotron Radiation Facility, Shanghai Institute of Applied Physics, Chinese Academy of Sciences, Shanghai 201204, China

## Abstract

The significant use of platinum for catalyzing the cathodic oxygen reduction reactions (ORRs) has hampered the widespread use of polymer electrolyte membrane fuel cells (PEMFCs). The construction of well-defined electrode architecture in nanoscale with enhanced utilization and catalytic performance of Pt might be a promising approach to address such barrier. Inspired by the highly efficient catalytic processes in enzymes with active centers embedded in charge transport pathways, here we demonstrate for the first time a design that allocates platinum nanoparticles (Pt NPs) at the boundaries with dual-functions of conducting both electrons by aid of polypyrrole and protons *via* Nafion^®^ ionomer within hierarchical nanoarrays. By mimicking enzymes functionally, an impressive ORR activity and stability is achieved. Using this brand new electrode architecture as the cathode and the anode of a PEMFC, a high mass specific power density of 5.23 W mg^−1^_Pt_ is achieved, with remarkable durability. These improvements are ascribed to not only the electron decoration and the anchoring effects from the Nafion^®^ ionomer decorated PPy substrate to the supported Pt NPs, but also the fast charge and mass transport facilitated by the electron and proton pathways within the electrode architecture.

Due to their high energy conversion efficiency and low environmental impact, PEMFCs are one of the most promising substitutes for batteries in portable devices and for internal combustion engines in vehicles^1^,^2^. However, the high cost of platinum for catalyzing the cathodic ORR has hampered the widespread use of PEMFC for these purposes^3^,^4^. The harsh acidic working conditions in PEMFCs limit the selection of electrocatalysts^2^,^5^. Pt is well-known as the most effective catalyst for both the anodic hydrogen oxidation reaction (HOR) and the cathodic ORR^6^. The ORR kinetics is six or more orders of magnitude slower than HOR, and thus considerable Pt loadings (0.2–0.4 mg cm^−2^) are required for the cathodic reaction^7^.

Enzymes, particularly metalloenzymes, demonstrate high activity towards many energy transformation reactions in biological systems, including ORR^8^. These natural catalysts usually consist of redox active metal centers where the catalytic reactions occur accompanied by electron and proton transfer. The active centers are typically embedded in the outer-sphere environments of proteins with channels or pathways for the transport of proton, electron, substrate and product between the outer surface of enzymes and the active centers^9^. This outer coordination sphere structure is thought to accelerate the reaction processes by delivering protons on demand and synchronize proton and electron transfers. Further, the interactions between the active sites and the adjacent amino acid residues are also thought to be critical to enhance the enzyme activity. Additionally, the outer spheres could also protect the fragile catalyst centers from degradation^10^. To catalyze the typical proton coupled electron transfer (PECT) reaction of ORR^11^, the over-potentials for the enzymes of bilirubin oxidase, laccase *et al.* are observed much smaller even than that of pure platinum surfaces^9^. The exceptional catalytic properties of enzymes were further exploited and integrated in energy conversion applications^12^,^13^,^14^. Learning from the functions of enzymes, it is possible to construct nano-scaled electrode architecture with active sites (e.g. Pt nano-particles) anchored or surrounded by charge carriers. Such design should be a promising approach to achieve enhanced electrode performance and durability, especially for the cathodic ORR involving not only the transfer of charges, but also the transport of gaseous reactants and liquid products^15^.

However, traditional electrode fabrications *via* painting of catalyst ink physically mixed with ionomers cannot effectively achieve such active centers within the electrode. The randomly distributed catalyst particles, proton carriers (ionomers) and pores rarely match with each other to ensure the smooth proceeding of the reactions. Recently, electrodes with spatially oriented arrays were designed to facilitate the mass transport of reactants and products^16^,^17^,^18^,^19^,^20^,^21^,^22^,^23^,^24^, promoting greater Pt utilization compared to the traditional disordered electrodes and reducing the total Pt loading to below 0.2 mg cm^−2^ (meeting commercialization requirements^4^). Although considerable achievements have been made on creating advanced electrode architecture, most of the current work focuses only on how to construct ordered mass transport channels, while the miro-environment coupled with proton and electron conductors have been largely neglected.

Therefore, to mimic the functions of enzymes with active centers embedded in transportation pathways, we propose a nano-architectural electrode with Pt nano-particles (NPs) anchored on the boundaries of transport channels for charges and mass, as schemed in Fig. 1. Briefly, combinational electron and proton pathways are constructed with vertically aligned nanowire arrays. To construct the nanowire arrays, Nafion^®^ decorated polypyrrole (NfnPPy) is grown directly on a gas diffusion layer (GDL) of fuel cells. In this configuration, the polypyrrole (PPy) acts as the electronic conductor, and the sulfonate group clusters of the Nafion^®^ ionomer act as the protonic conductor with the interspaces between the nanowires acting as continuous channels for reactant and product transport. Then, Pt NPs are anchored at the proton-conductive domains *via* strong chemical bonding to form the enzyme-like active sites for ORR. With such a nano-engineered electrode, both the Pt utilization and the durability of fuel cell are enhanced significantly. To our knowledge, this is the first attempt to design and construct such an electrode with nano-architecture tailoring to an electrochemical reaction.

## Results

### Structural Characterizations

The fabrication of this novel electrode, depicted in Fig. 2a–c, involves three major steps. The first step involves electrochemical polymerization of pyrrole decorated with Nafion^®^ ionomers to get ordered NfnPPy arrays on a GDL substrate. In the second step Pt ions are deposited on hydrophilic domains which are surrounded by sulfonate groups of Nafion^®^ ionomers at the surface of the ordered NfnPPy. This process is driven by the electrostatic interactions between Pt cations and sulfonate groups. Finally, the third step involves reducing Pt ions by gaseous hydrogen to form Pt NPs.

Polypyrrole, as a member of conjugated heterocyclic conducting polymers, presents great potential in fuel cell applications with relatively high electronic conductivity and electrochemical durability^25^. As shown in Fig. 2, vertically aligned nanowire array structures with an average diameter of 67 nm and an average length of 1.2 μm for a single PPy/Nafion hybrid wire are observed in FESEM images (Fig. 2d–f). Efficient doping Nafion^®^ ionomers into the PPy nanowires is confirmed by both the strong F signal in the XPS spectra (Fig. S1) and the C-F bonds appeared in the FTIR spectra of the nanowires (Fig. S2). The uniform distribution of F and S, tracing from the Nafion^®^ ionomers, as shown in Fig. 3a,b in the EDX mapping, demonstrates the homogeneous doping of Nafion^®^ in the PPy nanowires. In addition, the diffraction peak at 22.3° (attributed to the semi-crystalline structure of PPy^26^) in the XRD pattern of Nafion^®^ decorated PPy (Fig. S3) is broadened compared with that of the pure PPy. This suggests that the pristine long-range ordered PPy matrix is most likely partially destructed due to the modification of Nafion^®^ ionomers. Therefore, the above characterization analysis is consistent with extensive reports on molecular doping of polypyrrole in the literature^27^,^28^ in which Nafion^®^ ionomers doped into the matrix of PPy molecular chains creates a bi-conductor, that is, PPy as the electron conductor and Nafion^®^ ionomer as the proton conductor, as schemed in Fig. S4.

The anchoring of Pt ions, which were later reduced to Pt NPs by gaseous hydrogen, on the proton pathway is driven by the electrostatic interactions between Pt precursor cations and the sulfonate groups (with negative charges) of Nafion^®^ ionomers. This interaction will anchor the Pt NPs at the sites that are believed to play a role in the transfer of protons. The location of Pt NPs on the aligned NfnPPy nanowires is confirmed by analyzing the Pt distribution through the cross-section of the electrode *via* EDX as shown in Fig. S5. The Pt NPs with an average size of 4.3 nm are uniformly distributed on the surface of the decorated PPy nanowires, as demonstrated by the TEM images (Fig. 2g,h). The HRTEM image in Fig. 2i clearly indicates that the Pt NPs are well-defined spherical nano-crystallines. They are identified by the interplanar distance of 2.3 Å which is indexed to the lattice spacing of the Pt {111} planes. The particle size is consistent with the concept that the Pt NPs are located in the hydrophilic domains, which are constructed by the sulfonate groups^29^. A control experiment using PPy nanowires without a Nafion^®^ ionomer doping as the substrate produces smaller and more sparsely populated Pt NPs on the PPy nanowire surface, as shown in Fig. S6. This experiment further confirms the important role of the Nafion^®^ ionomer in the formation of uniform Pt NPs. To further understand the intrinsic interactions between Pt NPs and the sulfonate groups, we carried out XPS and XAS analysis of the Pt-NfnPPy sample. Interestingly, the Pt 4f_7/2_ peak for the Pt-NfnPPy shifts positively compared with that for the Pt-C (Fig. 3e), indicating that the outer-layer electrons of Pt are partially transferred. Accordingly, the S 2p spectrum for the Pt-NfnPPy, as shown in Fig. 3f, clearly presents broadened peaks ranging from 161 to 165 eV, assigned to S with lower valences than S (VI) in sulfonate groups of Nafion^®^ ionomer. This result strongly suggests that electrons transfer from Pt to S in the sulfonate groups^30^,^31^. Additionally, further analysis on the coordination of Pt in the Pt-NfnPPy is investigated *via* X-ray absorption fine structure spectroscopy (XAFS). To understand the intrinsic causality of the interactions between Pt and the sulfonate groups, the precursor of the Pt-NfnPPy, *i.e.*, the Pt(II)-impregnated NfnPPy (denoted as Pt(II)-NfnPPy), is also characterized. As shown in Fig. 3g, for the normalized X-ray absorption near-edge structure spectra (XANES) of the two samples, the white line (WL) intensity for Pt L_3_-edge are between those of PtO_2_ and Pt foil. Furthermore, the spectrum for Pt(II)-NfnPPy is much closer to the reference spectrum of PtO_2_ and the one for the Pt-NfnPPy is much closer to that of Pt foil. These results indicate that there is a high oxidation state of Pt in Pt(II)-NfnPPy but a low oxidation state of Pt in Pt-NfnPPy. To further explore the local coordination structure of Pt, quantitative information is extracted by the extended X-ray absorption fine structure (EXAFS) analysis as shown in Fig. 3h and the fitting results are in Table S1. Obviously, for Pt-NfnPPy, the peak around 1.7 Å (attributed to Pt-O) significantly declines compared with that of Pt(II)-NfnPPy, while the peak of Pt-Pt contribution rises. The fitting results in Table S1 further prove that the Pt-Pt metal bond (distance 2.75 Å, coordination number 5.7) exists in the nano Pt cluster of Pt-NfnPPy. Moreover, an enlargement in bond distance for Pt-O in sample Pt(II)-NfnPPy and Pt-NfnPPy (2.07 Å) is observed from Table S1, compared with that of the standard PtO_2_ sample (2.01 Å). This aberrant Pt-O bond that existed in the as-prepared samples indicates alternative chemical bonding between Pt and the sulfonate groups.

**Figure 1:**
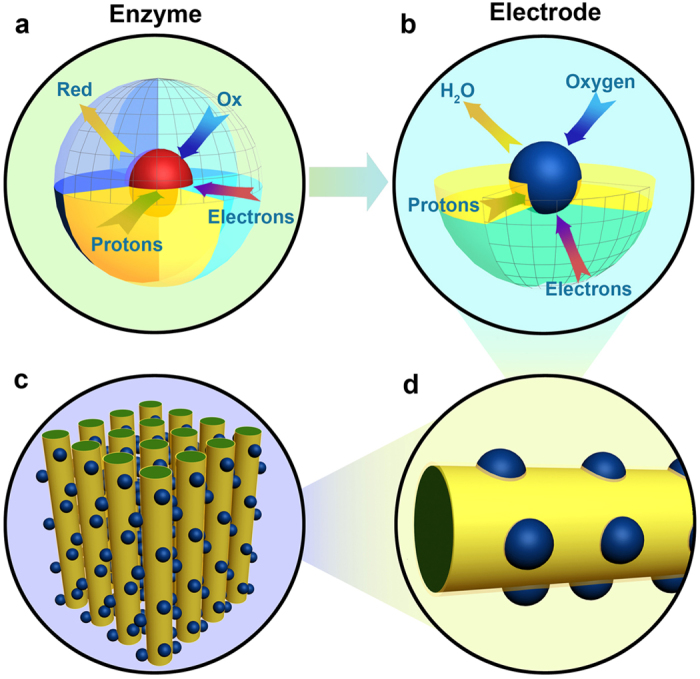
Schematic of the Pt-NfnPPy electrode inspired by the function of enzyme. (**a**) A typical structure of enzyme with active center embedded in the pathways for charges and mass. (**b**) Zoomed-in view of a Pt NP embedded in the substrate of NfnPPy. The PPy sunbstrate acts as electronic conductor, and the Nafion^®^ phase acts as protonic carrier. Oxygen and water can be transported through the ordered channels constructed by the nanowire arrays. (**c**,**d**) Pt-NfnPPy with hierarchical ordered structure.

**Figure 2:**
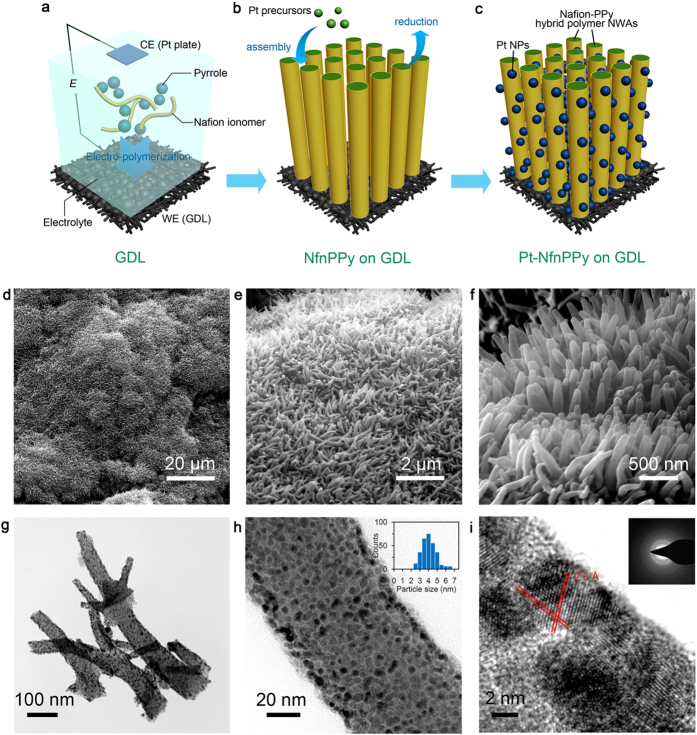
Schematic of the fabrication and the morphologies of the electrode arrays. (**a–c**) Synthetic scheme of the electrode arrays *via* electrochemical polymerization (**a**) Pt loading (**b**) and MEA fabrication (**c**). (**d–f**) SEM images of Pt-NfnPPy on the surface of a GDL at different magnifications. The rough surface of Pt-NfnPPy in a low magnification (**d**) represents the surface morphology of GDL built up with carbon powder. (**g**,**h**) TEM images of Pt-NfnPPy at different magnifications. Inset histogram of (**h**) is the Pt NP size distribution with an average size of 4.3 nm. (**i**) HRTEM image of Pt-NfnPPy with inset patterns of SAED.

**Figure 3:**
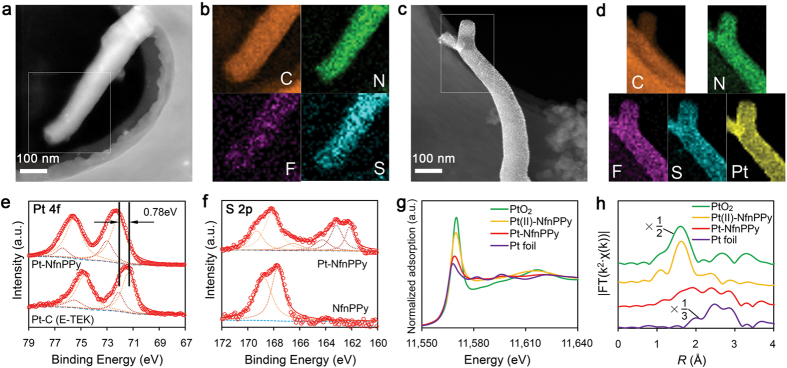
Elemental and structural analyses of NfnPPy and Pt-NfnPPy. (**a–d**) HAADF-STEM images (**a**,**c**) and the EDX mapping (**b**,**d**) of the marked areas. The elemental mapping of F and S in NfnPPy (**b**) represents the doping of Nafion^®^. After Pt loading, the tracing of Pt in Pt-NfnPPy mapping (**d**) follows the similar tracing of F and S. (**e**,**f**) XPS spectra of Pt 4f (**e**) and S 2p (**f**). Compared with commercial Pt-C (E-TEK), a positive shift of the peak attributed to metallic Pt is observed. (**g**) The normalized XANES spectra at the Pt L3 edge of sample Pt(II)-NfnPPy, Pt-NfnPPy, PtO_2_ and Pt foil. A decreasing trend of WL intensity is shown as PtO_2_ > Pt(II)-NfnPPy > Pt-NfnPPy > Pt foil. (**h**) The *k*^*3*^-weighted Fourier transform spectra from EXAFS for Pt(II)-NfnPPy, Pt-NfnPPy, PtO_2_ and Pt foil. The peaks at ~1.7 Å in the first shell are fitted to the Pt-O contribution, and the peaks at ~2.5 Å in the second shell are fitted to the Pt-Pt contribution.

The structures of Nafion^®^ anchored Pt are investigated using density functional theory (DFT, see Note S1 for more details). As illustrated in Table S2, the optimized structure of the Pt-sulfonate group demonstrates that a Pt atom (9Pt) is chemically bonded with two oxygen atoms (2O and 3O) of a sulfonate group. The calculated distance between 9Pt and 2O or 3O is simulated as 2.230 Å, which is much larger than the theoretical distance of Pt-O in PtO_2_ (2.040 Å). This is consistent with the results of the elongation of Pt-O bond distance (R) in the EXAFS results. Such a chemical bond between Pt and the sulfonate groups in Nafion^®^ ionomers could undoubtedly anchor the Pt NPs on the bi-conductor surface. Therefore, from the above analyses and the synthetic procedures, we can deduce that the Pt NPs are anchored in the hydrophilic domains surrounded by sulfonate groups of Nafion^®^ ionomers. This bond within the electrode architecture is expected to not only anchor the Pt NPs, but also potentially boost the proton transport, as designed in Fig. 1.

### Electrochemical characterizations and PEMFC single cell tests

**Figure 4:**
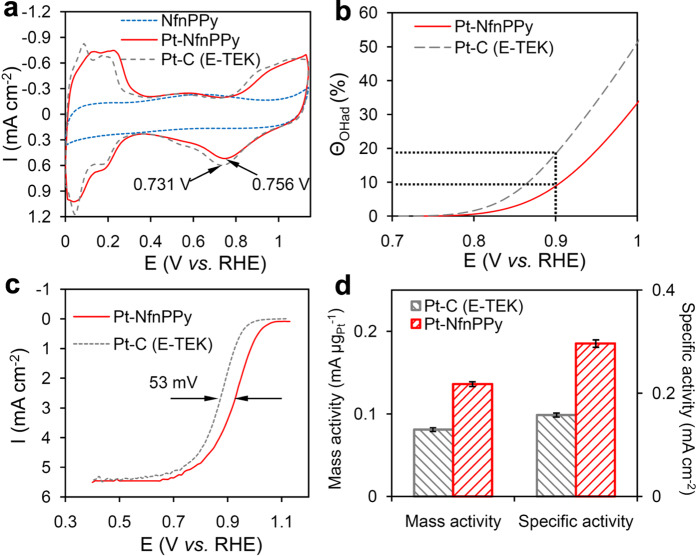
Electrochemical properties of Pt-NfnPPy and commercial Pt-C (E-TEK). (**a**) CV curves obtained in 0.5 M H_2_SO_4_ solution saturated by N_2_ at a scan rate of 50 mV s^−1^. (**b**) Hydroxyl surface coverage (Θ_OH_) results derived from the CV curves. (**c**) ORR polarization curves obtained in 0.5 M H_2_SO_4_ solution saturated by O_2_ at a scan rate of 10 mV s^−1^ and electrode rotation speed of 1600 rpm. (**d**) Mass activity and specific activity at 0.9 V (*vs.* RHE).

A typical pattern of hydrogen under-potential adsorption and desorption (H-UPD) peaks on Pt is observed from the CV curve after depositing Pt NPs on NfnPPy (Fig. 4a). The electrochemical active surface area (ECSA) could be calculated as 45.9 m^2^ g_Pt_^−1^ for Pt-NfnPPy by assuming a charge transfer of 210 μC cm^−2^ Pt surface^32^, which is similar to that of commercial Pt-C (E-TEK) (51.4 m^2^ g_Pt_^−1^). Taking into account of the particle size issue, the geometric surface area (GSA) of Pt particles in Pt-NfnPPy with an average diameter of 4.3 nm is calculated to be 65.2 m^2^ g_Pt_^−1^, presenting a higher Pt utilization (ratio of ECSA to GSA) of 70.4% than that of Pt-C (E-TEK) (53.1%). Additionally, the peak attributed to Pt oxide reduction of Pt-NfnPPy is located at 0.756 V *vs.* RHE, which is more positive than that of Pt-C (E-TEK) (0.731 V *vs.* RHE), revealing a weakening of the bonds between oxygen containing species and the Pt surfaces, which possibly comes from the electron modification of the substrate^33^. In agreement of this result, the fractional coverage by OH_ad_ (Θ_OHad_) of sample Pt-NfnPPy is reduced at the potential range over 0.7 V (*vs.* RHE) compared with that of Pt-C (E-TEK), as shown in Fig. 4b. Such property of lower OH_ad_ coverage might contribute to increasing the ORR activity and stability^34^,^35^. The half-wave potential of ORR polarization curve for Pt-NfnPPy exhibits an obviously positive shift of 53 mV compared with that of Pt-C (E-TEK) (Fig. 4c), indicating a higher electrochemical activity towards ORR. Mass activity and specific activity at 0.9 V *vs.* RHE of Pt-NfnPPy also exhibit superior values of 0.136 mA μg_Pt_^−1^ and 0.296 mA cm^−2^ (Fig. 4d), which are 1.7-fold and 1.9-fold greater than that of Pt-C (E-TEK), respectively.

**Figure 5:**
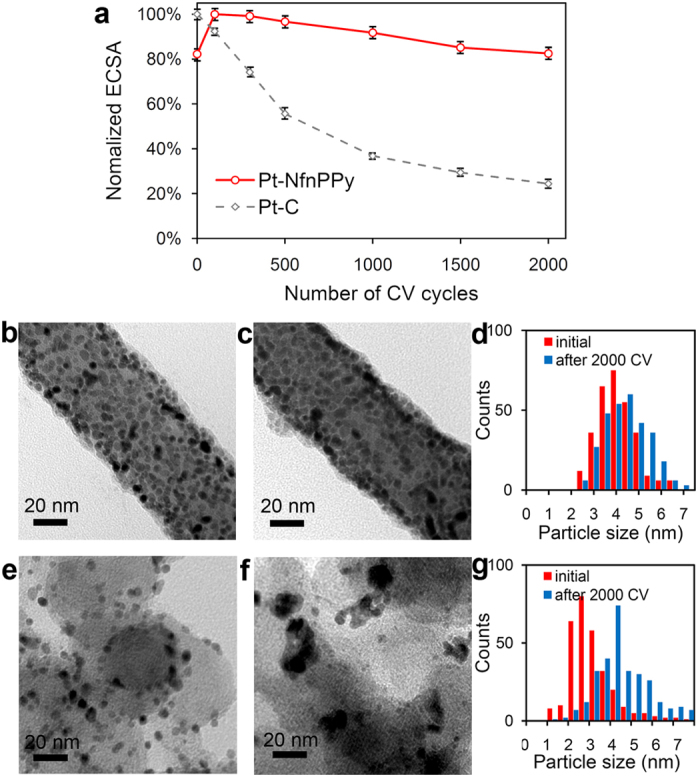
Electrochemical durability of Pt-NfnPPy and commercial Pt-C (E-TEK). (**a**) Normalized ECSAs of Pt after different cycles of ASTs. (**b**,**c**) TEM images of Pt-NfnPPy before (**b**) and after (**c**) ASTs. (**e**,**f**) TEM images of Pt-C (E-TEK) before (**e**) and after (**f**) ASTs. (**d**,**g**) Corresponding histogram of particle size distribution of Pt.

Further, the accelerated stress tests (ASTs) of Pt-NfnPPy and Pt-C (E-TEK) are conducted by applying linear potential sweeps between 0.6 and 1.2 V vs. RHE to estimate the electrochemical durability^36^. ECSA loss after 2000 cycles calculate from the CV curves (Fig. S7) in Pt-NfnPPy is only 18.3%, whereas the degradation for that of Pt-C (E-TEK) is much more severe with 80.4%, as shown in Fig. 5a. The ORR activity of Pt-NfnPPy also have a similar less decrease of 6 mV after ASTs, *vs.* 28 mV for that of Pt-C (E-TEK), as shown in Fig. S8a,b. Tracing back to the morphological change of the catalyst, we find out a much less aggregation of Pt NPs after ASTs (Fig. 5b–d) compared with the commercial sample (Fig. 5e–g), which indicates an enhanced stability brought by the anchoring effect of the substrate.

**Figure 6:**
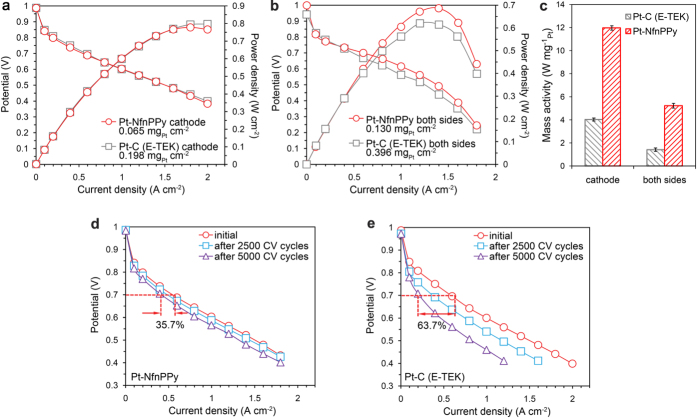
PEMFC performance and durability. (**a**) Polarization curves using a fully humidified H_2_/O_2_ supplement and maintained at 70 °C. (**b**) Polarization curves for PEMFCs equipped with Pt-NfnPPy and conventional Pt-C electrode as both sides of a MEA using a fully humidified H_2_/air supplement and maintained at 70 °C. (**c**) Mass specific power density for PEMFCs equipped with Pt-NfnPPy and conventional Pt-C electrode. (**d**,**e**) Polarization curves after different ASTs cycles for Pt-NfnPPy (**d**) and conventional Pt-C (**e**) cathode.

To demonstrate the functions imitated from enzymes in practical applications of this hierarchically nano-architectural electrode, we used a piece of Pt-NfnPPy (with a Pt loading of 0.065 μg cm^−2^) hot-pressed to a Nafion^®^ 112 membrane to obtain a MEA for PEMFC tests. As shown in Fig. 6a, the open circuit potential for PEMFC with Pt-NfnPPy is similar to that of a single cell using commercial Pt-C (E-TEK) catalyst with a three-time Pt loading (0.198 mg cm^−2^). This result benefits from a better electrochemical activity towards ORR for Pt-NfnPPy sample compared with Pt-C. The peak power density of the single cell is up to 0.778 W cm^−2^, comparable to that of a conventional cathode fabricated by commercial Pt-C (E-TEK) with a much higher Pt loading. In addition, this performance indicates a significant enhancement of the Pt utilization in this novel cathode, which is further identified by the increased ECSA in MEA (Table 1). The mass specific power density of the cathode also reaches up to 11.97 W mg^−1^_Pt cathode_, which is 3.0-fold higher than that of the conventional cathode (Fig. 6c). The Pt-NfnPPy is also attached to both sides of a PEMFC and tested under the DOE’s reference conditions, as shown in Fig. 6b. The mass specific power density of the newly structured single cell reaches 5.23 W mg^−1^_Pt total_, which is 3.7-fold greater than the commercial catalyst coating and exceeding the DOE’s target for 2015 (5 W mg^−1^_Pt total_).

The durability of the novel electrode is tested by the ASTs conducted at the cathode under practical working conditions of a fuel cell. As shown in Fig. 6d, the polarization curve for the PEMFC with Pt-NfnPPy exhibits a mitigated degradation rate compared with a commercial Pt-C cathode as the potential cycling number increased. The current density at 0.7 V for the PEMFC with Pt-NfnPPy degrades 35.7% after 5000 cycles, whereas a remarkable loss of 63.7% for the conventional cathode with Pt-C is observed (Fig. 6e). Consistent with the degradation trend of the fuel cell discharge currents, the ECSA loss in the cathode exhibits a similar tendency. For example, a 41.7% degradation occurs in the ECSA for the Pt-NfnPPy sample, whereas a 63.5% degradation occurs for the Pt-C (E-TEK) sample (Fig. S9). Additionally, long-term operation durability is tested by performing a 100-hour lifetime test at the current density of 0.5 A cm^−2^ for a single cell with Pt-NfnPPy equipped on both sides (Fig. S10), and also demonstrates a remarkable performance.

### Elucidation on the remarkable performance of Pt-NfnPPy

Such high catalytic activity, Pt utilization and durability achieved in both a half cell and a single cell is probably ascribed to the elaborate design of an enzyme-resembled electrode architecture, in which simultaneous electron/proton transfer could occur around Pt NPs anchored by the sulfonate group clusters. Hence, to explore the intrinsic causalities of the enhanced performance over current electrodes, we carry out further experimental and theoretical studies on the functions imitated from enzymes. As a typical proton coupled electron transfer (PCET) reaction, ORRs occurring on the surfaces of Pt NPs can be boosted by the enhanced electron/proton transport. The electronic conductance of NfnPPy is measured as ~25 S cm^−1^, which is sufficient for electrode applications^20^,^37^,^38^. The surrounded sulfonate group clusters perform as the outer coordination spheres of enzymes to accelerate the transportation of protons. In order to validate such function, the protonic conductivity of the NfnPPy layer is measured by a two-electrode method as schemed in Fig. S11a. Noticeably, as shown in Fig. S11b, the protonic conductivity of NfnPPy is much higher than that of the pure PPy at the testing temperatures (323–363 K), and also exceeds that of the traditional Nafion^®^ ionomer bonded catalyst layer at the working temperature of fuel cells (343–363 K). Additionally, an increase in conductivity for NfnPPy with temperature is similar to that observed for Nafion^®^ membranes, indicating an identical mechanism of proton transport in NfnPPy as that in continual hydrophilic domains formed by the Nafion^®^ ionomers^29^.

**Figure 7:**
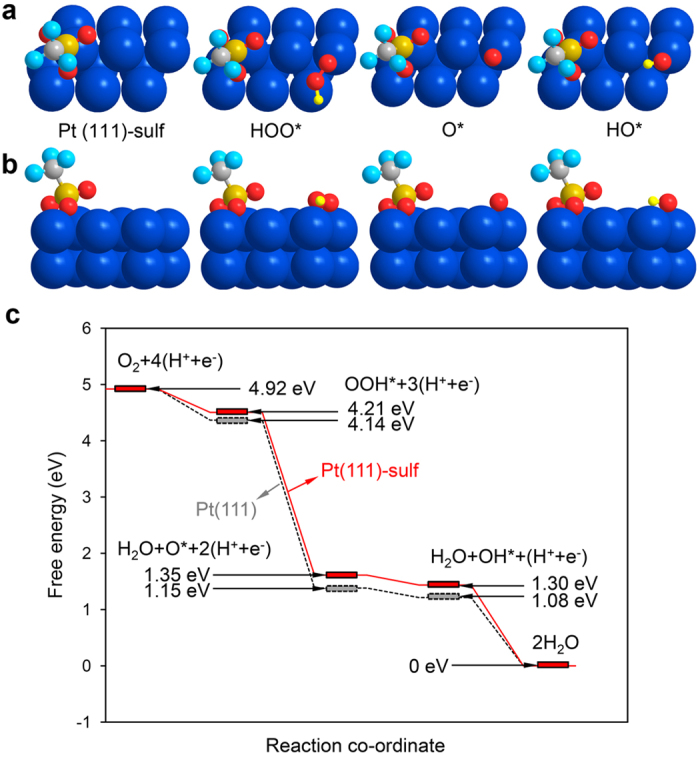
DFT calculation results. (**a**,**b**) Top (**a**) and side (**b**) view of sulfonate group anchoring on Pt (111) surface, and the intermediates forming of ORR. (**c**) Free-energy diagram for the corresponding reaction steps on Pt (111) surface with (red solid line) and without (grey dash line) the anchoring of a sulfonate group.

The chemical bonding between the Pt NPs and the surrounding sulfonate group might be another critical factor for the enhanced ORR activity and durability. Hence, we carried out extensive theoretical calculations using DFT. Based on the theoretical results above, we constructed a 3 × 2 Pt (111) surface with a sulfonate group anchored on (Fig. 7). As a critical descriptor for ORRs, the binding energy of atomic oxygen and hydroxyl is calculated on such a modified Pt (111) surface, with the results shown in Table S3. Compared with a pure Pt (111) surface, the sulfonate group modified one exhibits a slight decrease in absorption energy of atomic oxygen (−4.06 eV) and hydroxyl (−2.11 eV). This result might predict to yield an optimized catalytic activity towards ORR according to numerous studies previously^39^. Based on the calculated data, the intermediates adsorbed on the sulfonate group decorated Pt (111) surface is illustrated in Fig. 7a,b, and the corresponding free-energy diagram is also presented below (Fig. 7c). These changes in adsorption energy could ascribe to the electron modification of sulfonate groups towards Pt, as shown in the XPS results. Furthermore, the anchoring effect between sulfonate groups and Pt surfaces could be the reason for the enhanced stability of Pt NPs, which is also a similarity for the burying of active centers in enzyme structures.

The experimental and theoretical results together identify the enzyme-like functions towards ORRs brought by the hierarchical nano-architecture of Pt-NfnPPy. The micro environment of proton/electron pathways surrounding Pt NPs, which acts as the functions brought by the outer coordination spheres of enzymes, could greatly improve the electrochemical performance and stability in two major aspects. Firstly, the electron decoration of sulfonate groups significantly accelerates the processes of ORR with weakened intermediate adsorption. Secondly, the enhanced proton transport, associated with well coupled electron and mass transport pathways, greatly boosts the electrochemical processes within the MEA under work conditions. Furthermore, the applications of this novel nano-architectural design could be extended to other reactions involving electron/proton transfer and gas/liquid transport.

## Discussion

In this work we proposed a brand new enzyme-inspired and generally illuminating design of an electrochemical electrode with high performance and durability. The key point of this electrode architecture is to anchor and stabilize active sites (Pt NPs) in the environment constructed by proton/electron pathways. Such nano-environment for electrochemical reactions located in the highly oriented nanowire arrays with smooth mass transport channels. Our straightforward electrochemical method successfully creates an ordered nano-structure with Pt NPs anchored on the clusters of Nafion^®^ ionomer decorated PPy nanowires *via* the strong chemical bonding between Pt NPs and the sulfonate groups. Such well-defined structure for an electrode provides increased transport and reaction regions for PEMFC cathode processes and consequently demonstrates a high cell performance with a remarkable cathode mass specific power density of 11.97 W mg^−1^_Pt cathode_, which is 3.0-fold greater than that of a conventional cathode with commercial Pt-C catalyst. This novel electrode is applied to both sides of a MEA and demonstrated an impressive performance of 5.23 W mg^−1^_Pt total_, which exceeds the DOE’s target for 2015. We believe that the functional mimic of enzymes presented in this work has applications in many next-generation power devices, e.g. fuel cells, lithium ion batteries, super capacitors and metal-air batteries. Additionally, an enhanced understanding of the “micro-environment” in physical and chemical aspects for electrochemical reactions could be further developed based on this concept for an electrode.

## Methods

### Chemicals and Synthesis

NfnPPy was fabricated on the substrate of a GDL (SGL 35BC carbon paper) *via* electrochemical polymerization with a typical potential applied to the working electrode of 0.65 V (*vs.* SCE) for 45 min. The electrolyte consisted of 0.2 M phosphate buffered solution, 0.1 M *p*-toluenesulfonyl sodium, 0.1 M pyrrole and 0.1 wt% Nafion^®^ ionomer (10 wt% dispersed in water, Sigma Aldrich). A piece of GDL, a saturated calomel electrode (SCE) and a Pt plate were used as the working electrode (WE), the reference electrode (RE) and the counter electrode (CE), respectively. A typical loading of NfnPPy on the GDL was 0.2 mg cm^−2^. The samples of PPy nanowire arrays without Nafion^®^ decorated (denoted as PPy) were synthesized the same as the other samples without adding Nafion^®^ ionomer.

Pt nano-particles (NPs) were allocated *in situ* on the NfnPPy coated GDL *via* electrostatic interactions. Typically, a piece of NfnPPy coated GDL was immerged in an aqueous solution of 0.03 M Pt(NH_3_)_2_(NO_2_)_2_ for 48 hours at 70 °C. Afterwards the samples were washed by de-ionized water three times. Then, the NfnPPy coated GDL with Pt ions was reduced in hydrogen at 250 °C for 4 hours, and the as-prepared sample was denoted as Pt-NfnPPy. The Pt loading was determined *via* X-ray fluorescence (XRF, Thermo QUANT’X) spectrometry as 0.065 ± 0.007 mg cm^−2^. For comparison, the sample with Pt deposited on the pure PPy followed the same procedures and was denoted as Pt-PPy.

### Physical Characterization

The as-prepared samples were analyzed *via* X-ray diffraction (XRD, D/max-2400X, Ricoh) spectra, Fourier transform infrared (FTIR) spectroscopy (Nicolet 6700, Thermo Fisher), field emission scanning electron microscopy (FESEM, JSM-6360LV, JEOL) and transmission electron microscopy (TEM, JEM-2011EM, JEOL).

The X-ray photoelectron spectroscopy (XPS) analyses were performed using a Kratos AMICUS spectrometer equipped with a monochromatic Mg X-ray source (Mg-Kα, 1.2536 keV). High-resolution elemental analyses were performed on the C 1s (295–275 eV), N 1s (390–410 eV), O 1s (545–525 eV), S 2p (160–180 eV) and Pt 4f (84–64 eV) regions with a pass energy of 20 eV, a 0.05 eV step and an 800 ms dwell time. Each spectrum was constructed from an average of two scans. The pressure in the XPS analysis chamber was maintained at 10^−7^ Pa or lower during collection. In the data analysis, the binding energy (BE) of the core level C 1s peak was set at 284.5 eV to compensate for surface-charging effects. The Shirley background was subtracted, and the satellite peaks were removed for all element peaks before curve fitting. The experimental spectra were fitted into components of a Gaussian line shape.

High resolution TEM (HRTEM), selected area electron diffraction (SAED), high angle annular dark field scanning transmission microscopy (HAADF-STEM), energy dispersive X-ray (EDX) spectra and mapping were carried out with a Tecnai G2 20 microscope (FEI).

Pt L_3_-edge X-ray adsorption spectroscopy (XAS) was performed on the BL14W1 beamline using Si (111) double-crystal monochromator at the Shanghai Synchrotron Radiation Facility (SSRF), Shanghai Institute of Applied Physics (SINAP), China, operated at 3.5 GeV with injection currents of 210 mA. Pt foil and PtO_2_ were used as reference samples. All the samples were measured in the transmission mode.

### Electrochemical Characterization

The electrochemical characterization was conducted in a three-electrode cell with a CHI 760D potentiostat/galvanostat at room temperature (298 K). The sample of Pt-NfnPPy mixed with carbon powder from MPL was peeled off from the GDL substrate for electrochemical testing with a typical amount of 0.485 mg cm^−2^_GDL_. The electrodes for electrochemical tests were prepared by loaded catalyst materials onto the glassy carbon electrode with a area of 0.196 cm^2^. The loading of Pt-NfnPPy and commercial 20 wt% Pt-C (E-TEK) were controlled to be 62.5 and 51.0 μg_Pt _cm^−2^, respectively. 0.5 M H_2_SO_4_ solution, a SCE and a Pt wire was used as electrolyte, RE and CE, respectively. Cyclic voltammetry (CV) between 0 and 1.14 V *vs.* RHE at a scan rate of 50 mV s^−1^ was performed under the continuous purging of high purity N_2_. ORR polarization curves were obtained by CV tests between 0 and 1.14 V *vs.* RHE at a scan rate of 10 mV s^−1^ in oxygen saturated electrolyte. The accelerating stress tests (AST) were conducted *via* CV cycling between 0.6 and 1.2 V *vs.* RHE at a scan rate of 100 mV s^−1^ in N2 saturated 0.5 M H_2_SO_4_ electrolyte.

### MEA Fabrication and Single Cell Tests

A piece of Nafion^®^ 212 membrane (DuPont) was used as the ion-conducting membrane. A piece of commercial Pt loaded GDL (Sunrise Power Co.) was used as an anode, while the cathode was the as-prepared Pt-NfnPPy coated GDL. The membrane electrode assemblies (MEAs) with an active area of 2 × 2 cm^2^ were fabricated by hot pressing the membrane sandwiched between the anode and the cathode of Pt-NfnPPy coated GDL at 120 °C and 200 kg cm^−2^ for 1 min. A control MEA sample was fabricated by using a cathode sprayed commercial Pt-C (E-TEK) with a Pt loading of 0.198 mg cm^−2^. Particularly, the commercial catalyst was mixed with 20 wt% Nafion^®^ ionomer (5 wt% solution, DuPont^®^), 15 times de-ionized water and 15 times ethanol by ultrasonication for 30 min. The as prepared ink was sprayed on a piece of GDL at the substrate temperature of 60 °C. Then the cathode was hot pressed with the same anode and membrane mentioned above to fabricate the control MEA sample. All the MEAs were inserted into graphite end plates with serpentine gas flow channels to assemble single cell units.

The PEMFC performance and durability was evaluated *via* a Fuel Cell Test Station (Fuel Cell Technologies, Inc.) at a cell temperature of 70 °C with full humidity. At the DOE’s reference condition, the polarization curves were obtained at a total outlet pressure of 150 kPa on both sides. The anode side was fed with hydrogen with a stoichiometry of 2, and the cathode side was fed with oxygen or air with a stoichiometry of 9.5 or 2 respectively. At the dead-end mode condition, the anode side was fed with hydrogen with a stoichiometry of 2 without back pressure, and the cathode side was fed with oxygen with a stoichiometry of 1 with the outlet closed. The CV curves between 0 and 1.2 V were obtained at the same temperature with a scan rate of 50 mV s^−1^. The anode side was fed with hydrogen with a flow rate of 100 mL min^−1^ used as RE and CE, and the cathode side was fed with nitrogen with a flow rate of 200 mL min^−1^ used as WE. The durability was characterized *via* performing similar ASTs with sweeping CV cycles between 0.6 and 1.2 V, at a scan rate of 100 mV s^−1^.

## Additional Information

**How to cite this article**: Xia, Z. *et al.* Bio-inspired Construction of Advanced Fuel Cell Cathode with Pt Anchored in Ordered Hybrid Polymer Matrix. *Sci. Rep.* **5**, 16100; doi: 10.1038/srep16100 (2015).

## Supplementary Material

Supplementary Information
