# Supplementary material for: Bio-inspired Construction of Advanced Fuel Cell Cathode with Pt Anchored in Ordered Hybrid Polymer Matrix
Source: Sci Rep. 2015 Nov 5;5:16100. doi: 10.1038/srep16100 (PMC4633593; doi:10.1038/srep16100)
Supplement: Supplementary Information [file srep16100-s1.pdf]

Electronic Supplementary Information

Bio-inspired Construction of Advanced Fuel Cell Cathode with Pt Anchored in Ordered Hybrid Polymer Matrix

Zhangxun Xia, Suli Wang, Luhua Jiang, Hai Sun, Shuang Liu, Xudong Fu, Bingsen Zhang, Dang Sheng Su, Jian-Qiang Wang*, and Gongquan Sun**

Note S1. DFT calculation details.

The DFT calculations for the structure of Pt anchored by Nafion® ionomers were preformed with the Gaussian 03 program. The three-parameter hybrid functional of Becke (B3LYP) was used with the exchange term described by the exchange functional of Becke and the nonlocal correlation functional described by the LYP expression[1, 2]. The Los Alamos LANL2DZ effective core pseudopotential (ECP) and the corresponding valence double-ζ basis set was adopted for Pt atoms, whereas the 6-311+G** basis set was used for all C, O, F and S atoms[3, 4]. We carried out full geometry optimization with different geometries and spin states. The geometry with the lowest energy was regarded as the ground state and was considered for the further discussion. The calculation parameters for the ground state were listed in Table S2.

The DFT calculations for the intermediates forming on the sulfonate group anchored Pt (111) surfaces were performed using the Vienna ab-initio simulation package (VASP)[5-8], where ultrasoft pseudopotential was used to describe the ionic cores. The Kohn–Sham one-electron valence states were expanded in a plane-wave basis set with kinetic cutoff at 340 eV. The exchange-correlation energy and potential were described by the generalized gradient functional self-consistently, GGA-PW91[9, 10]. During iterative diagonalization of the Kohn–Sham Hamiltonian, Fermi population of the Kohn–Sham states (kBT = 0.1 eV) and Pulay mixing of the resulting electronic density was used to improve the convergence, and the total energy is extrapolated to absolute zero correspondingly.

Table S1. EXAFS parameters of Pt foil, PtO_2, Pt(II)-NfnPPy and Pt-NfnPP.

Sample	Path	N^a	R^b	σ^2 (10^{-3} Å^2)^c	ΔE_0 (eV)^d
Pt foil	Pt-Pt	12	2.76 ± 0.01	4.4 ± 0.1	6.0 ± 0.7
PtO_2	Pt-O	6	2.01 ± 0.01	3.0 ± 0.5	11.7 ± 0.7
	Pt-Pt	2	3.07 ± 0.02	2.2 ± 0.7	7.5 ± 5.3
Pt(II)-NfnPPy	Pt-O	3.4 ± 0.4	2.07 ± 0.02	6.0	7.6 ± 2.8
Pt-NfnPPy	Pt-O	1.1 ± 0.2	2.07 ± 0.01	4.0	13
	Pt-Pt	5.7 ± 0.9	2.75 ± 0.01	7.9 ± 1.0	6.4

a Coordination number; b Distance between absorber and back scatterer atoms; c Debye–Waller factor; d Inner potential correction.

Table S2. Structure parameters for the interaction between Pt atom and the sulfonate group of Nafion® ionomer cluster calculated by DFT.

**Cluster**	**Mulliken charge**	***d*** **(Å)**	**Electron number**
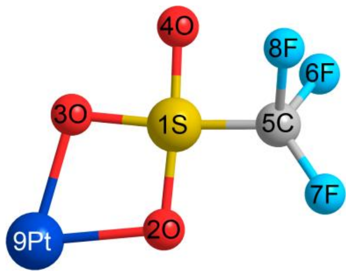	1S 1.065		
	2O -0.525	1S-2O: 1.523	1S-2O: 0.237
	3O -0.525	1S-3O: 1.523	1S-3O: 0.236
	4O -0.405	1S-4O: 1.443	1S-4O: 0.453
	5C 0.461	9Pt-1S: 2.808	9Pt-1S: -0.099
	6F -0.159	9Pt-2O: 2.230	9Pt-2O: 0.101
	7F -0.158	9Pt-3O: 2.230	9Pt-3O: 0.101
	8F -0.167	9Pt-4O: 4.046	9Pt-4O: 0.006
	9Pt 0.413		

Table S3. Adsorption energy of the intermediates on the surface of pure Pt (111) and sulfonate group anchored Pt (111) calculated by DFT.

**Surfaces**	**Adsorption energy (eV)**		
	O*	HO*	HOO*
Pt (111)	-4.288	-2.330	-1.160
Pt (111)-sulf	-4.059	-2.111	-1.089

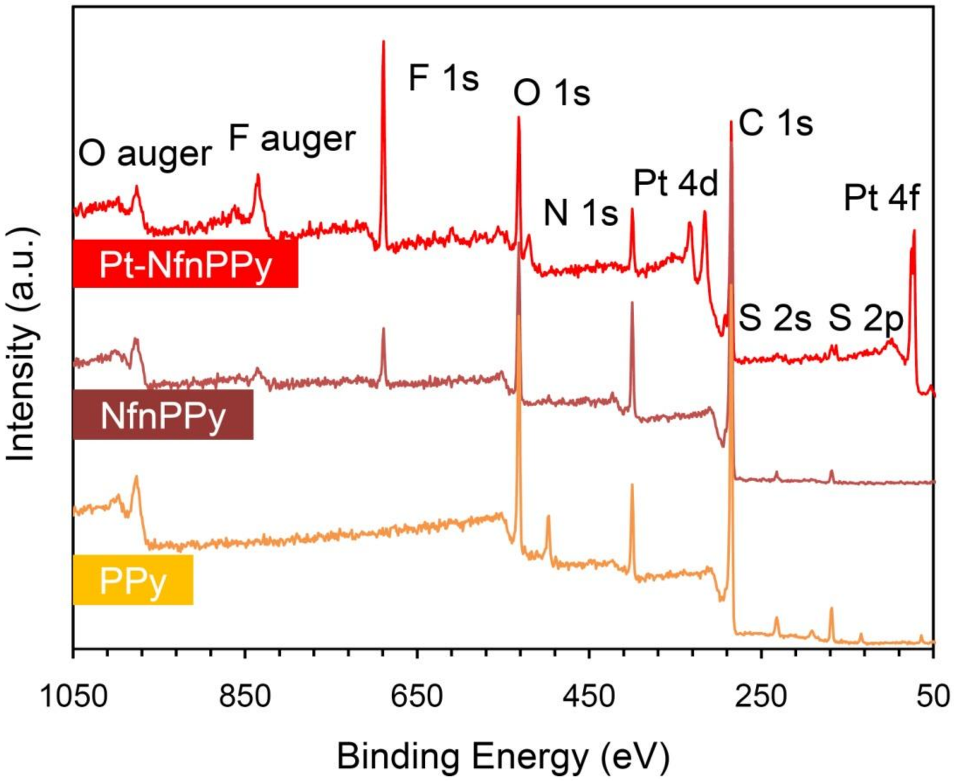

Figure S1. XPS survey spectra for Pt-NfnPPy, NfnPPy and PPy. The signals due to O 1s, N 1s, C 1s, S 2s and S 2p can be attributed to the PPy nanowires with the shape directing molecules. The doping of Nafion® ionomer is confirmed by the signals of F 1s in the spectra of Pt-NfnPPy and NfnPPy, and the loading of Pt is seen in the peaks of Pt 4d and Pt 4f in the spectrum of Pt-NfnPPy.

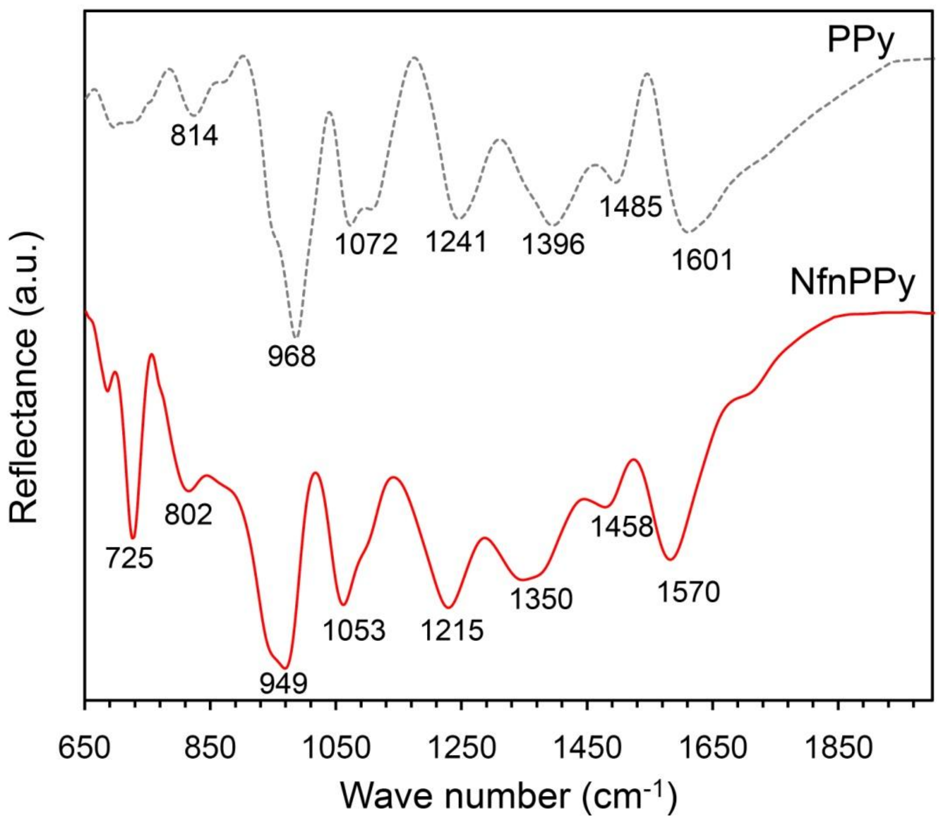

Figure S2. FTIR spectra for NfnPPy and PPy. The peaks locating at around 814, 968, 1072, 1241, 1396, 1485 and 1601 cm^{-1} are associated with PPy, and a strong peak located at 725 cm^{-1} confirms the existence of C-F bonding.

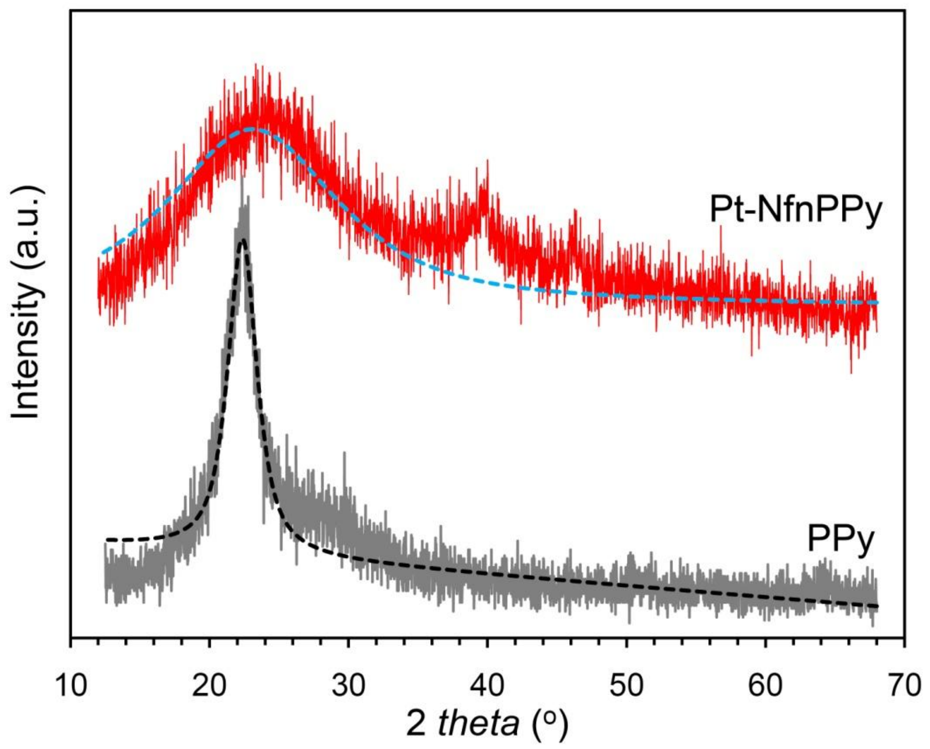

Figure S3. XRD patterns for Pt-NfnPPy and PPy. The peak located at around 22.3°, which is attributed to the semi-crystalline structure of PPy, is obviously broadening after doping Nafion® ionomers.

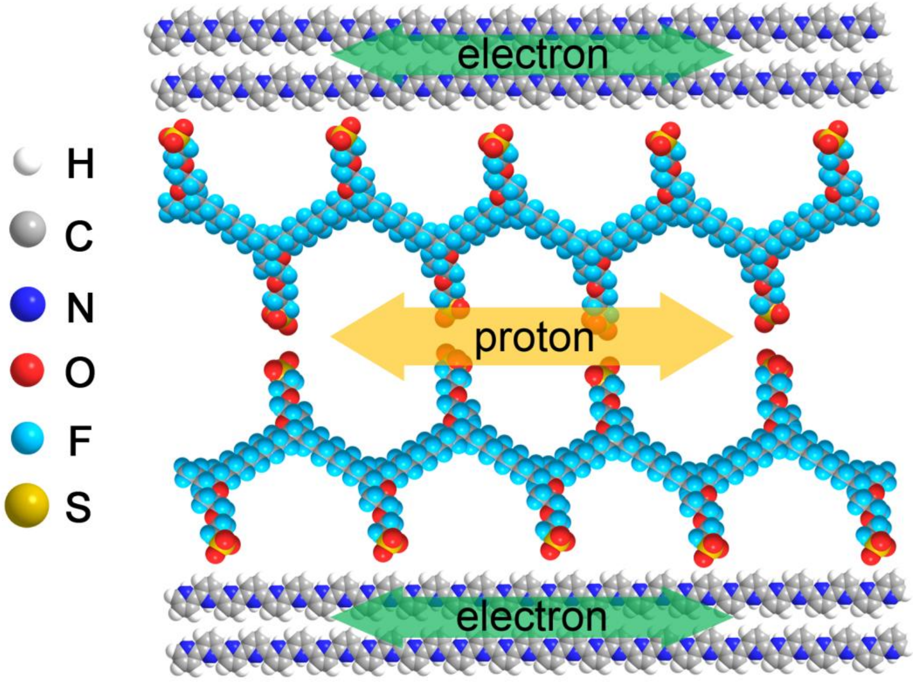

Figure S4. Schematic of the matrix of the bi-conductor polymer. Polypyrrole chains possess the electronic conductivity derived from the π-conjugation structure. The proton channels can form from the continual clusters of the hydrophilic side chains of Nafion® ionomers.

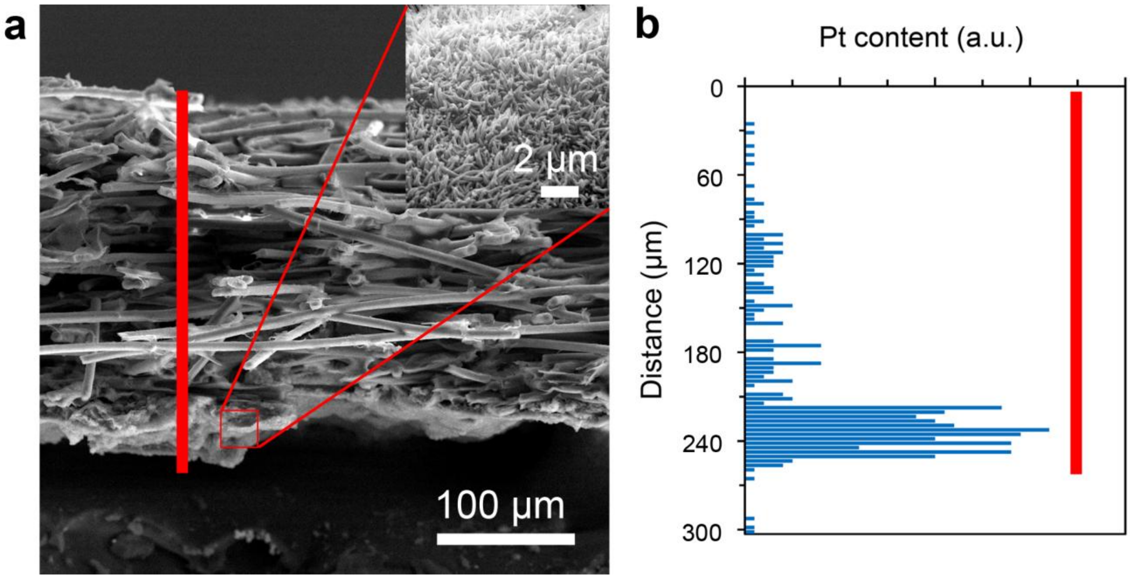

Figure S5. Pt distribution over the cross-section of the gas diffusion electrode. a) SEM images in the cross-section view of the gas diffusion electrode. The Pt-NfnPPy layer is on the top of the MPL as shown in the insert top-view image marked with the red square. b) EDAX analysis of Pt distribution over the red line marked in the SEM image (a). The Pt is largely deposited within the layer of NfnPPy, which suggests that the ion-exchange process is the major course for Pt loading.

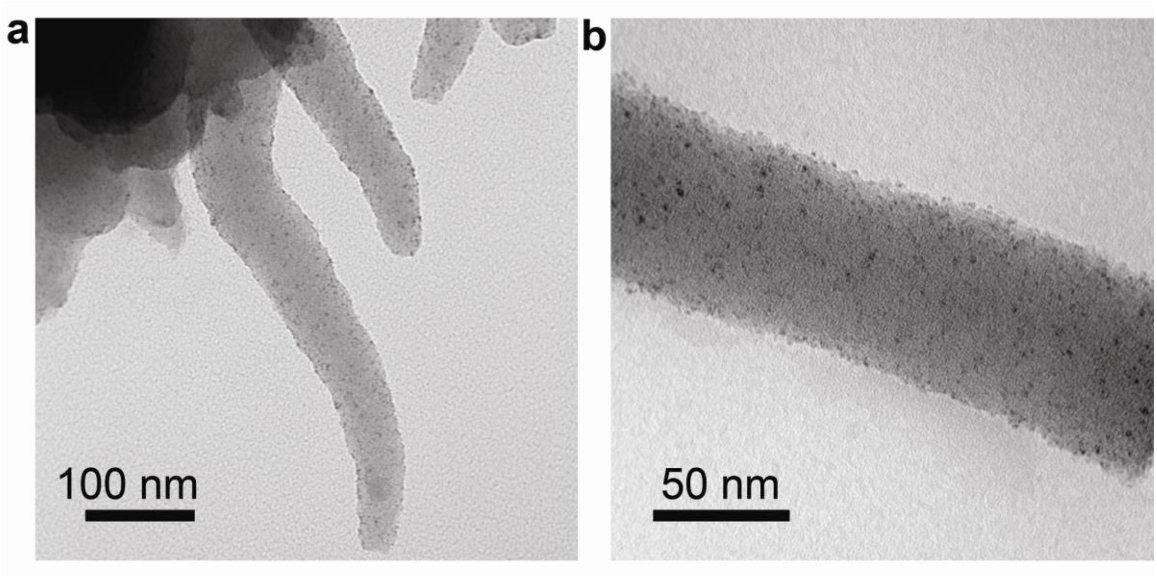

Figure S6. TEM images of Pt-PPy. Similar nanowire structure is formed in the absence of Nafion® ionomers (a). However, Pt NPs is only around 1~2 nm and sparse in the absence of Nafion® ionomers (b).

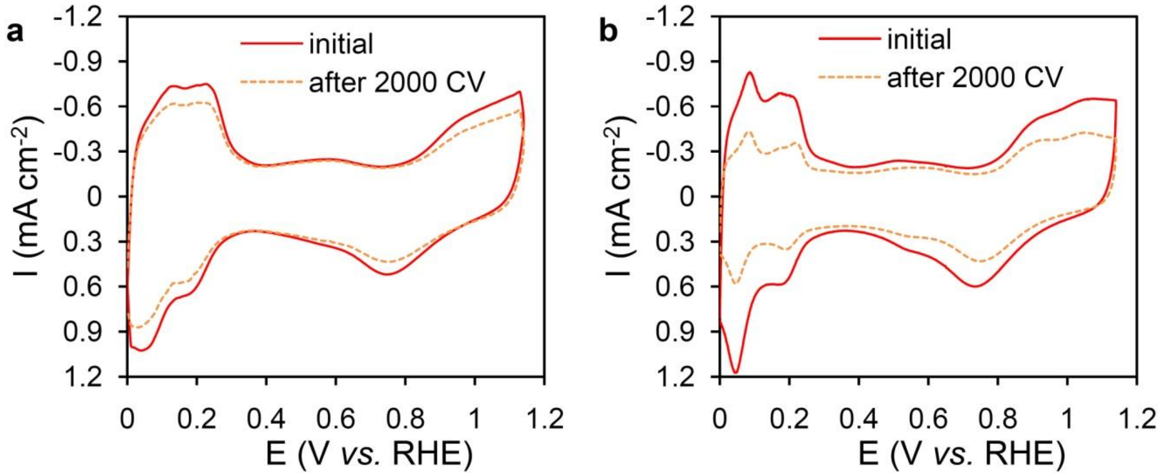

Figure S7. CV curves before and after ASTs for Pt-NfnPPy (a) and Pt-C (E-TEK) (b).

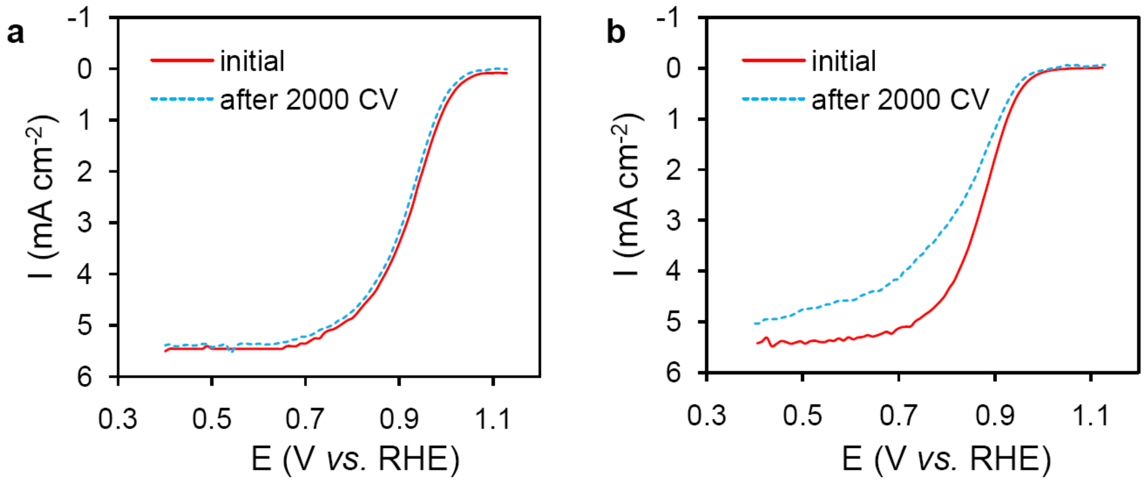

Figure S8. ORR polarization curves before and after ASTs for Pt-NfnPPy (a) and Pt-C (E-TEK) (b).

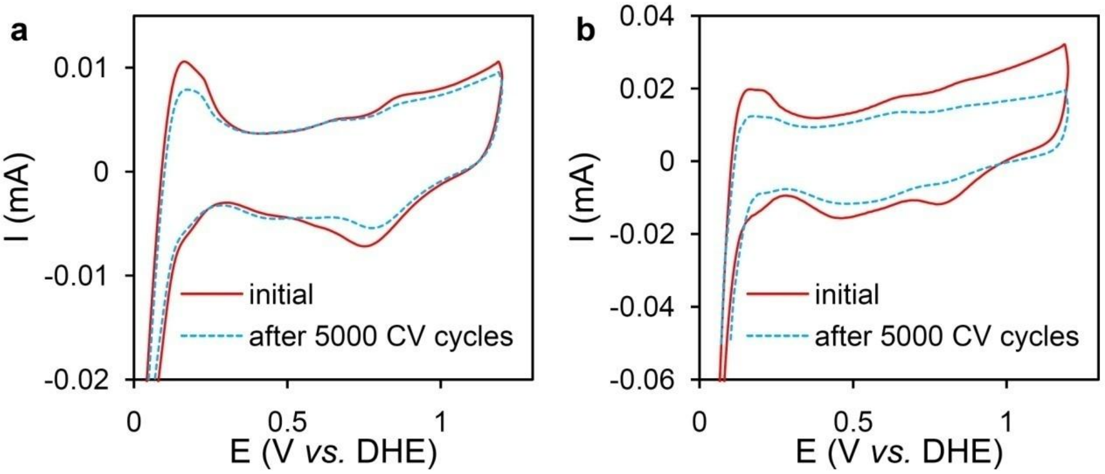

Figure S9. CV curves before and after ASTs for cathodes with Pt-NfnPPy (a) and Pt-C (E-TEK) (b).

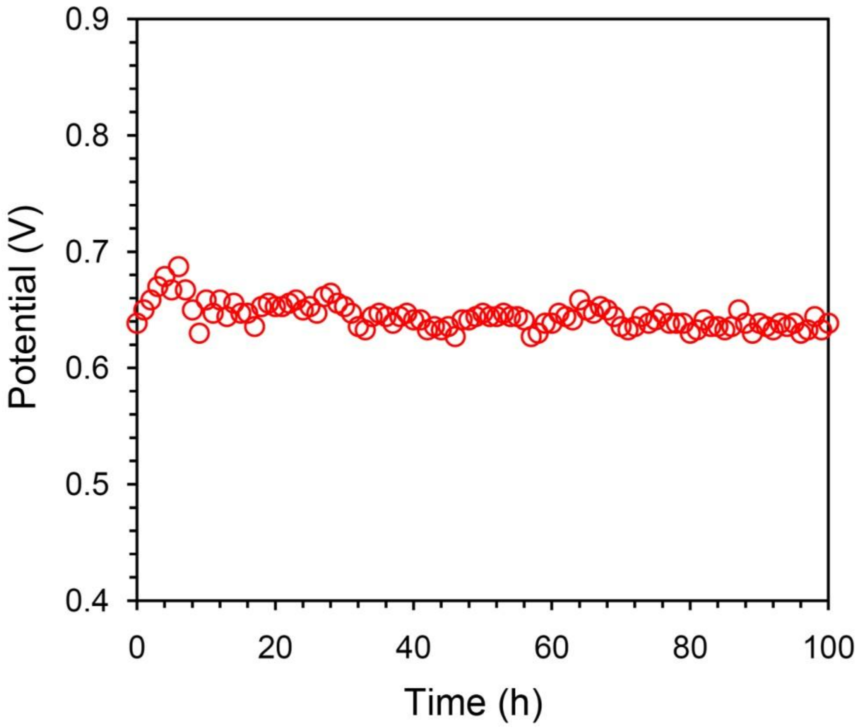

Figure S10. Life-time test of the PEMFC equipped with Pt-NfnPPy on both sides of MEA at a current density of 0.5 A cm^{-2}. The anode and cathode were fed with a fully humidified H_2/O_2 supplement with a stoichiometry of 2 and 9.5 respectively at total outlet pressure of 150 kPa and maintained at 70 ^oC.

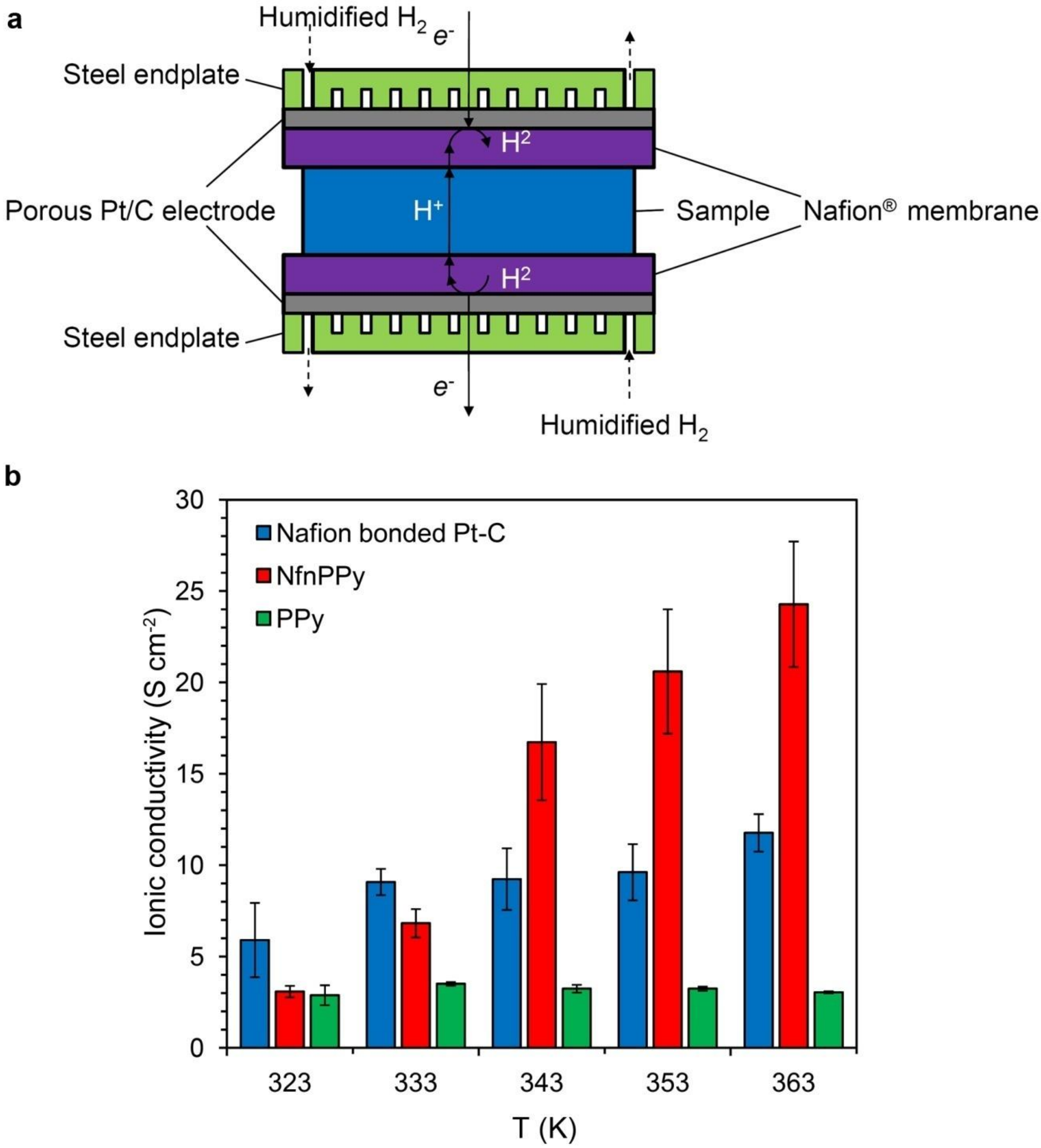

Figure S11. (a) Mearsurement of ionic conductivity for the samples with combined electronic and ionic conductance. (b) Ionic conductivity tested under different temperature.

References

1. A. D. Becke, *J. Chem. Phys.*, 1993, 98, 5648.
2. C. Lee, W. Yang and R. G. Parr, *Phys. Rev. B*, 1988, 37, 785.
3. P. J. Hay and W. R. Wadt, *J. Chem. Phys.*, 1985, 82, 270.
4. P. J. Hay and W. R. Wadt, *J. Chem. Phys.*, 1985, 82, 299.
5. G. Kresse and J. Hafner, *Phys. Rev. B*, 1993, 47, 558.

6 G. Kresse and J. Hafner, *Phys. Rev. B*, 1994, 49, 14251.

7 G. Kresse and J. Furthmuller, *Comput. Mater. Sci.*, 1996, 6, 15.

8 G. Kresse and J. Furthmuller, *Phys. Rev. B* 54, 1996, 11169.

9 J. P. Perdew, J. A. Chevary, S. H. Vosko, K. A. Jackson, M. R. Pederson, D. J. Singh and C. Fiolhais, *Phys. Rev. B*, 1992, 46, 6671.

10 J. A. White and D. M. Bird, *Phys. Rev. B*, 1994, 50, 4954.
